# Transformation of detritus by a European native and two invasive alien freshwater decapods

**DOI:** 10.1007/s10530-018-1661-z

**Published:** 2018-01-31

**Authors:** Thomas M. Doherty-Bone, Alison M. Dunn, Caroline Liddell, Lee E. Brown

**Affiliations:** 1 School of Biology, University of Leeds, Leeds, West Yorkshire UK; 2 School of Geography, University of Leeds, Leeds, West Yorkshire UK; 3 water@leeds, University of Leeds, Leeds, West Yorkshire UK

**Keywords:** *Austropotamobius pallipes*, *Eriocheir sinensis*, *Pacifastacus leniusculus*, Shredding, FPOM, Decomposition rates, Invasive alien species, Ecosystem functioning

## Abstract

Invasive alien species have the potential to alter biodiversity and ecosystem processes. In freshwaters, detritus decomposition is a major ecosystem service but it remains uncertain whether invasive alien decapods process detritus differently to natives. This study examined leaf litter processing, and cascading effects on biofilms, by the European native white clawed crayfish (*Austropotamobius pallipes*) compared to two invasive alien decapod species: the American signal crayfish (*Pacifastacus leniusculus*) and the Chinese mitten crab (*Eriocheir sinensis*). Invasive alien decapods were responsible for higher leaf litter decomposition than the native. In comparison with native crayfish, invasive alien crab and crayfish showed higher rates of litter consumption, increased production of smaller leaf fragments, fine particulate organic matter (FPOM) and dissolved organic carbon. Nutrients (ammonia and soluble reactive phosphorous) derived from excretion (measured separately in the absence of biofilms) varied among decapod species, being lower for *P. leniusculus.* However, nutrient concentrations did not vary among species in the detritivory experiments with biofilm, implying nutrients were utilised for biofilm production and respiration as no differences in biomass were evident among decapod treatments. These results show invasive alien decapods have the potential to increase the magnitude of detrital processing to FPOM in rivers, but indirect impacts on primary producers due to nutrient release are uncertain based on this experimental context.

**Electronic supplementary material:**

The online version of this article (10.1007/s10530-018-1661-z) contains supplementary material, which is available to authorized users.

## Introduction

Invasive alien species are organisms translocated by human action from their native range to a biogeographically novel locality, where they become established and spread (Blackburn et al. 2011). Invasive alien species can alter community structure and modify ecosystem processes (Ehrenfeld 2010; Strayer 2012), especially in freshwater ecosystems (Strayer and Dudgeon 2010; Moorhouse and Macdonald 2015). One major freshwater ecosystem process is the recycling of leaf litter and its transformation into other forms of energy and nutrient throughout the food web (Cummins et al. 1973; Gessner et al. 2010). Freshwater decapods, particularly crayfish, are key processors of allochthonous riparian resources in their native ranges, with crabs studied to a lesser extent (Schofield et al. 2001; Rosewarne et al. 2013). A recent meta-analysis of the impacts of aquatic invasive species on lower trophic levels and ecosystem attributes found substantial impacts, including on nitrogen and organic matter standing stocks but did not explicitly examine detritus decomposition rates (Gallardo et al. 2015).

Invasive species often differ from taxonomically analogous natives in physiological and behavioural traits (e.g. Dick et al. 2014), and in their trophic position (Tran et al. 2015). Species vary in their body elemental composition, which co-varies with their consumption of resources (Vanni 2002). Invasive alien species thus have the potential to alter nutrient cycling through possessing novel stoichiometric traits (Capps and Flecker 2013), which can be indicated by nutrient composition of excreta. Decapod crustaceans excrete ammonia, soluble reactive phosphorous and other nutrients (Kristiansen and Hessen 1992). The fragmentation of leaf litter potentially also increases organic matter surface area for microbial activity. Released nutrients are then made available to primary trophic levels such as heterotrophic microbes and algae (Dyson et al. 2007; Kominoski et al. 2014).

Many studies on leaf litter processing by invasive alien decapods have looked only at the invasive species without comparing processing by invasives to the analogue native (Moore et al. 2012; Twardochleb et al. 2013), which may be because it has already been driven to extinction (e.g. Rudnick and Resh 2005; Schuster et al. 2010). In the British Isles and mainland Europe west of the Alps, the indigenous White Clawed Crayfish, *Austropotamobius pallipes* still maintains viable populations in some areas but is at risk of total extirpation with the advance of two larger invasive decapods: the American Signal Crayfish, *Pacifastacus leniusculus*, which can be followed by invasions of the Chinese Mitten Crabs (*Eriocheir sinensis*) (Rogers and Watson 2010; Almeida et al. 2014). If these invasive decapods differ in their leaf litter shredding function, this could have profound impacts on whole river basin resource processing rates and wider food webs.

This study investigated the impact of these native versus invasive alien freshwater decapod crustaceans on the processing of allochthonous resources (abscised leaf litter) and the consequences of this on a lower trophic level (biofilm). The study tested the following hypotheses: (H_i_) the two invasive alien species would consume and breakdown more leaf litter than the native crayfish, owing to their greater consumption and metabolic rates (Rosewarne et al. 2014, 2016); (H_ii_) Production of secondary products such as smaller leaf fragments (coarse particulate organic matter CPOM), fine particulate organic matter (FPOM) and dissolved nutrients would be higher in invasive alien species treatment due to their increased consumption, thus excretion rate; (H_iii_) enhanced production of secondary products in the invasive decapod treatments would have a positive impact on the biomass of biofilm, via dissolved nutrients fuelling growth.

## Methods

### Materials

Alder (*Alnus glutinosus*) leaves collected around the University of Leeds campus on senescence were oven dried at 50 °C and stored in paper bags prior to experimentation. Alder was chosen as it is a dominant native riparian species in Europe, and is commonly used in studies on decomposition rates (Abelho 2001). Leaves were subsequently combined into pre-weighed packs (3.0–3.5 g), placed in a labelled mesh bag, and conditioned (sensu Gessner et al. 1999) in water from a nearby stream (Meanwood Beck, Leeds: 53.820937N, 1.5604979W) for 2 weeks for microbial communities to colonise.

Chinese Mitten Crabs were obtained from the River Thames at Chiswick, London (51.488489N, 0.24471175E). American Signal Crayfish were collected from Fenay Beck, Huddersfield (53.641660N, 1.7310895W). White Clawed Crayfish were collected from Clapham Beck, North Yorkshire (54.117165N, 2.3921497W), Wyke Beck, West Yorkshire (53.827819N, 1.4893696W) and Adel Beck, West Yorkshire (53.855078N, 1.5743397W). All animals were captured by hand, held in the laboratory for a minimum of 2 weeks prior to experimentation for acclimatization, and fed Crab Cuisine^®^ pellets ad libitum. Animals were starved for 24 h prior to the experiment start to standardise hunger (Rosewarne et al. 2016).

To examine biofilm growth, unglazed stone tiles (22 × 22 × 10 mm, with a reactive surface area of 1364 mm^2^) were incubated outdoors for 3 months (July–October 2014) in a water tank seeded from a nearby lake (Wothersome Lake, Bramham; 53.874944N, 1.3913008W) and pond (Kirkstall Valley Nature Reserve, Leeds; 53.811316N, 1.6032428W). The tiles were then brought into a controlled environment room (see below) for 2 weeks to acclimatise before use in the experiments. A subset of 25 tiles were sampled to measure biofilm biomass at the start of the experiment.

### Experimental design

Microcosms were set up in the laboratory to compare the effects of native and invasive alien decapod species on leaf litter processing in a controlled environment. Microcosms consisted of 30 × 20 × 15 cm (4 l) plastic tanks containing dechlorinated tap water that was aerated throughout the study. Microcosm chambers consisted of an upper section containing leaf litter, a single decapod crustacean and a PVC pipe for shelter; and a lower chamber containing a single biofilm-colonized tile and another clean tile to measure growth of new biofilm during the experiment. The upper and lower chambers were separated by 1 mm aperture nylon mesh. The mesh allowed FPOM particulates < 1 mm to pass through whilst retaining larger leaf litter fragments in the upper chamber for further consumption. The mesh also served to isolate the lower chamber and accumulated FPOM and biofilm tile from the decapod. Microcosms were incubated at 14 °C on a 16:8 h light:dark cycle.

Four treatments were established: (1) White Clawed Crayfish, *A. pallipes* (native crayfish); (2) American Signal Crayfish, *P. leniusculus* (invasive alien crayfish); (3) Chinese Mitten Crab, *Eriocheir sinensis* (invasive alien crab); and (4) a control with no decapods, to measure the effects of microbial conditioning and (to a lesser extent, sensu Gessner et al. 1999) leaching. Sub-adult decapods (mass range 1.49–19.8 g) were size-matched as closely as possible among species (see Supp. Fig. 1) and added to microcosms following a randomised design. This age class forms a significant proportion of populations of all these species (Doherty-Bone personal observation) and been shown to shred leaf litter (Bondar et al. 2005; Rosewarne et al. 2013). All treatments consisted of 24 replicates (with equal representation of males and females), with the exception of Chinese Mitten Crabs (13 replicates; 6 males and 7 females; fewer individuals were available owing to mortality). The experiment ran for 14 days. On day 7, 4 l of water was exchanged through siphoning to ensure welfare of decapods (Kemp et al. 2003).

After 14 days, measurements were taken of: decapod mass, remaining leaf litter mass, mass of smaller coarse particulate organic matter fragments (CPOM, 1–10 mm), mass of fine particulate organic matter (FPOM, 0.7 µm–0.1 mm), nutrient concentrations (organic and inorganic carbon, ammonia, nitrate, soluble reactive phosphorous). To measure decomposition rates, CPOM and FPOM production, the contents of the upper chamber were rinsed in microcosm water by gently moving the mesh to ensure that all FPOM dropped to the lower chamber. Remaining leaf litter was placed in labelled paper bags. Smaller leaf fragments (CPOM; 1–10 mm) in the top layer of the microcosm were also collected using tweezers and placed in paper envelopes. Leaf litter and CPOM were dried at 50 °C, weighed and ashed at 500 °C to estimate ash-free dry mass (AFDM). Decomposition rate (AFDM per day) was calculated by subtracting final AFDM from the starting AFDM of leaf litter, following Benfield (2006). Fine particulate organic matter (FPOM) was sampled from a 50 ml aliquot of the homogenized microcosm water, filtered on a pre-ashed 0.7 µm GF/F filter disc, and ash-free dry mass estimated following Ramchunder et al. (2011).

To measure the response of dissolved nutrients, water samples were taken from the water column at the end of the experiment. A Skaler SAN ++ continuous flow auto-analyser was used to measure ammonia (NH_3_-N), nitrate (NO_3_-N) and soluble reactive phosphorous (PO_4_-P); an Analytik Jena Multi NC2100 combustion analyser was used to measure dissolved organic carbon (DOC). To compare dissolved nutrients derived from decapod nutrient-excretion in the absence of microbial biofilms on leaf litter and tiles (that would absorb nutrients), a separate incubation was set up for the same treatments as above (a decapod free control, *A. pallipes*, *P. leniusculus*, *E*. *sinensis*, *n* = 10 replicates per treatment) following modified protocols of Vanni et al. (2002). Decapods were unfed for 24 h and added to dechlorinated tap water in the same 4 l containers as above for 24 h, alongside control microcosms that had no decapod added over the same amount of time. After the 24 h incubation, water samples were taken and processed for dissolved nutrients (NH_3_-N, NO_3_-N, PO_4_-P) as described above.

To measure response of biofilm, tiles were removed and the biofilm extracted from each separately using a firm nylon brush rinsed with deionised water, making up to 50 ml solution. A 5 ml sub-sample of the homogenised slurry was then filtered on to pre-ashed GF/F filter discs (0.7 µm) and chlorophyll *a* extracted in dark conditions then measured using a portable spectrophotometer (Hach DR/2010) following Steinman et al. (1996). The remainder of the slurry was filtered as for FPOM, dried in an oven at 105 °C and ash free dry biomass (AFDM) estimated from loss on ignition at 500 °C (Steinman et al. 1996).

### Data analysis

All data were analysed using R (R v.3.1.0.; R Core Team 2014). Generalised Linear Models (formula: glm) were used to compare the response variables (leaf litter decomposition rate, detritivory performance, CPOM production, FPOM production, dissolved nutrients, biofilm productivity) against the different treatments. Mass varied among crabs and crayfish species despite size-matching (Supp. Fig. 1). Because mass can influence resource consumption, it was incorporated with decapod species into the GLMs, with the best fit model (species identity or mass) inferred using lowest value of Akaike’s Information Criterion (AIC) (Burnham and Anderson 2004). Post-hoc Tukey tests were used to test for differences among treatments. For those response variables that showed significant relationships, effect size was calculated using Cohen’s *d* (Cohen 1992). Effect sizes of 0–0.2 are interpreted to be negligible, 0.21–0.79 moderate and > 0.8 strong in either the negative or positive direction (Cohen 1992).

## Results

### Leaf litter decomposition

**Fig. 1:**
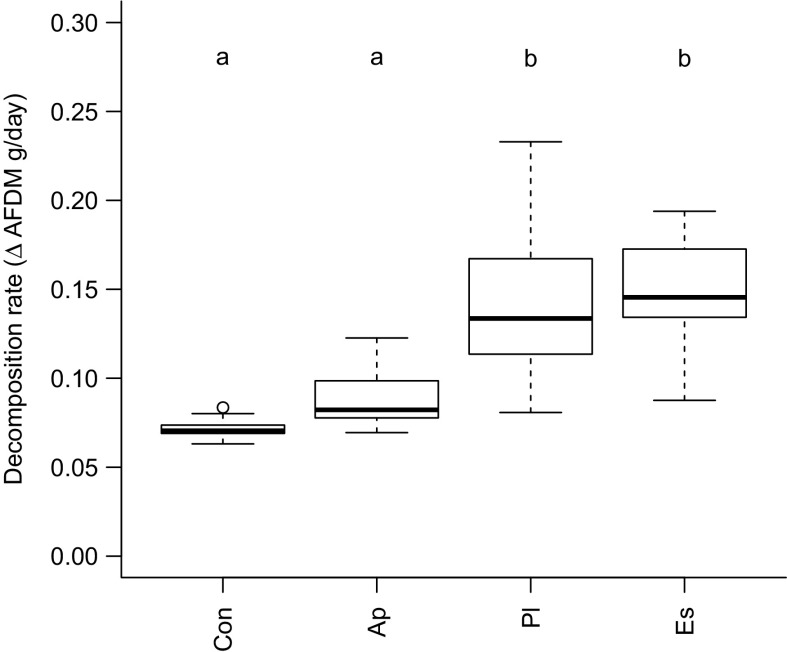
Processing of leaf litter quantified from measurements of decomposition rates (g loss of AFDM per day). Abbreviations on x-axes: Con, control; Ap, native crayfish (*Austropotamobius pallipes*); Pl, invasive alien crayfish (*Pacifastacus leniusculus*); Es, invasive alien crab (*Eriocheir sinensis*). Letters above boxplots indicate groupings based on post hoc tests

Leaf litter decomposition rate differed significantly among species (Table 1), being greater for the invasive aliens *E. sinensis* and *P. leniusculus* compared to the native *A. pallipes* and the control (Fig. 1). Effect sizes were large for all decapod treatments versus controls, but markedly higher for invasive alien compared to native species (Suppl. Fig. 3). There was a significant species–mass interaction (*p* = 0.001, Table 1), with *P. leniusculus* decomposition rate more strongly correlated with mass (*p* > 0.001, R^2^ = 0.42, slope coefficient = 0.28) than *A. pallipes* (*p* = 0.01, R^2^ = 0.15, coefficient = 0.08) (Suppl. Fig. 4). The best performing model however was with species alone as an explanatory factor (Table 1). As the size of the species differed slightly, detritivory performance was explored (g AFDM loss per day per mass of decapod). Detritivory performance showed the same relationship, differing significantly among species (Table 1; Suppl. Fig. 5), being higher for the invasive alien decapods. Change in mass of decapods during the experiment did not vary significantly among the species treatments (Table 1).

**Table 1:** Generalised linear models with experimental treatment as the factor

Hypothesis	Response variable	Effects model	*df*	Residual deviance	Pr(> Chi)	AIC
(1) Breakdown of leaf litter	Decomposition rate	**Species**	**3**	**0.087**	**<** **0.001**	**−** **379**
		Mass	1	0.033	< 0.001	− 240
		Species * mass	2	0.007	0.001	− 280
	% change in decapod mass	Species	2	626.54	0.368	537
		Mass	1	385.02	0.267	535
		Species * mass	2	769.58	0.286	538
	Detritivory performance	**Species**	**2**	**0.074**	**< 0.001**	
(2) Secondary products	CPOM (10–1 mm size) production	**Species**	**3**	**0.089**	**<** **0.001**	**−** **296**
		Mass	1	0.040	< 0.001	**−** 188
		Species * mass	2	0.012	0.036	− 201
	FPOM production	Species	3	33.168	< 0.001	169
		Mass	1	14.372	< 0.001	149
		Species * mass	**2**	**3.137**	**0.025**	**126**
	DOC production	Species	3	43.647	0.013	366
		**Mass**	**1**	**30.863**	**0.005**	**261**
		Species * mass	2	9.893	0.277	263
	Ammonia (NH_3_-N)	Species	3	0.012	0.249	− 245
		Mass	1	0.028	0.003	− 175
		Species * mass	**2**	**0.026**	**0.007**	**−** **181**
	Nitrate (NO_3_-N)	Species	3	0.581	0.536	134
		Mass	1	0.009	0.859	105
		**Species** * **mass**	**2**	**2.418**	**0.015**	**104**
	Soluble reactive phosphorous (PO_4_-P)	Species	3	0.082	0.1053	− 119
		**Mass**	**1**	**0.113**	**0.003**	**−** **86**
		Species * mass	2	0.038	0.246	− 81
(3) Biofilm response	Biomass accrual	Species	3	0.000	0.665	− 904
	Primary productivity	Species	3	0.2331	0.720	96
	Biomass of established tiles	Species	3	0.000	0.196	− 1274
	Primary productivity of established tiles	Species	3	1.612	0.347	178

Models that show lowest AIC value are highlighted in bold

### Secondary products from detritivory

**Fig. 2:**
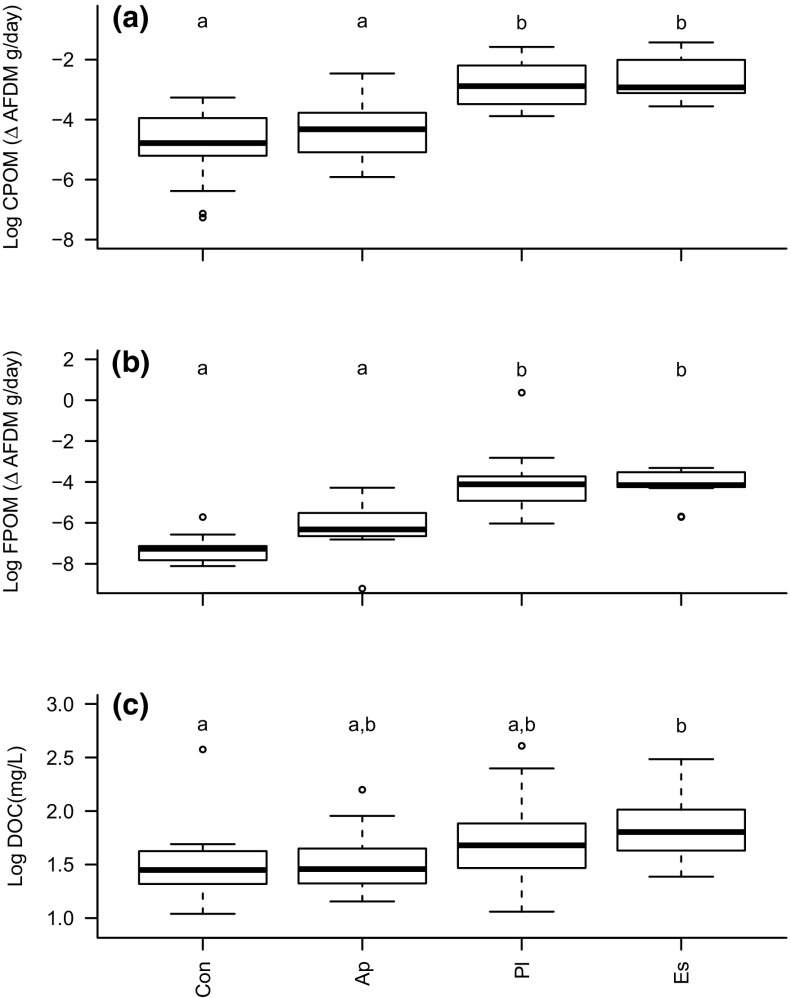
Products derived from detritivory. **a** log_10_ production of smaller fragments (CPOM 10–1 mm) (g AFDM per day), **b** log_10_ FPOM (1 mm–0.7 µm) production per day (g AFDM per day), **c** log_10_ dissolved organic carbon (mg/l). Abbreviations of x-axis and letters above box-plots as per Fig. 1. Letters above boxplots indicate groupings based on post hoc tests

Production of CPOM fragments was significantly influenced by decapod species, mass and the mass–species interaction (Table 1; Fig. 2a). As with decomposition rate, species identity showed the best performing model when compared to mass or the species–mass interaction. Both invasive alien species produced more CPOM compared to the native (Fig. 2a). Invasive species produced more FPOM than the native. There was also a significant species–mass interaction for FPOM, which showed the best performing model compared to species identity or mass alone (Fig. 2b; Table 1). Dissolved organic carbon concentration differed significantly among the species treatments with more produced by invasive decapods (Fig. 2c). DOC was affected by decapod mass (with lowest AIC value) but did not interact significantly with the species–mass interaction (Table 1).

### Dissolved nutrients

**Fig. 3:**
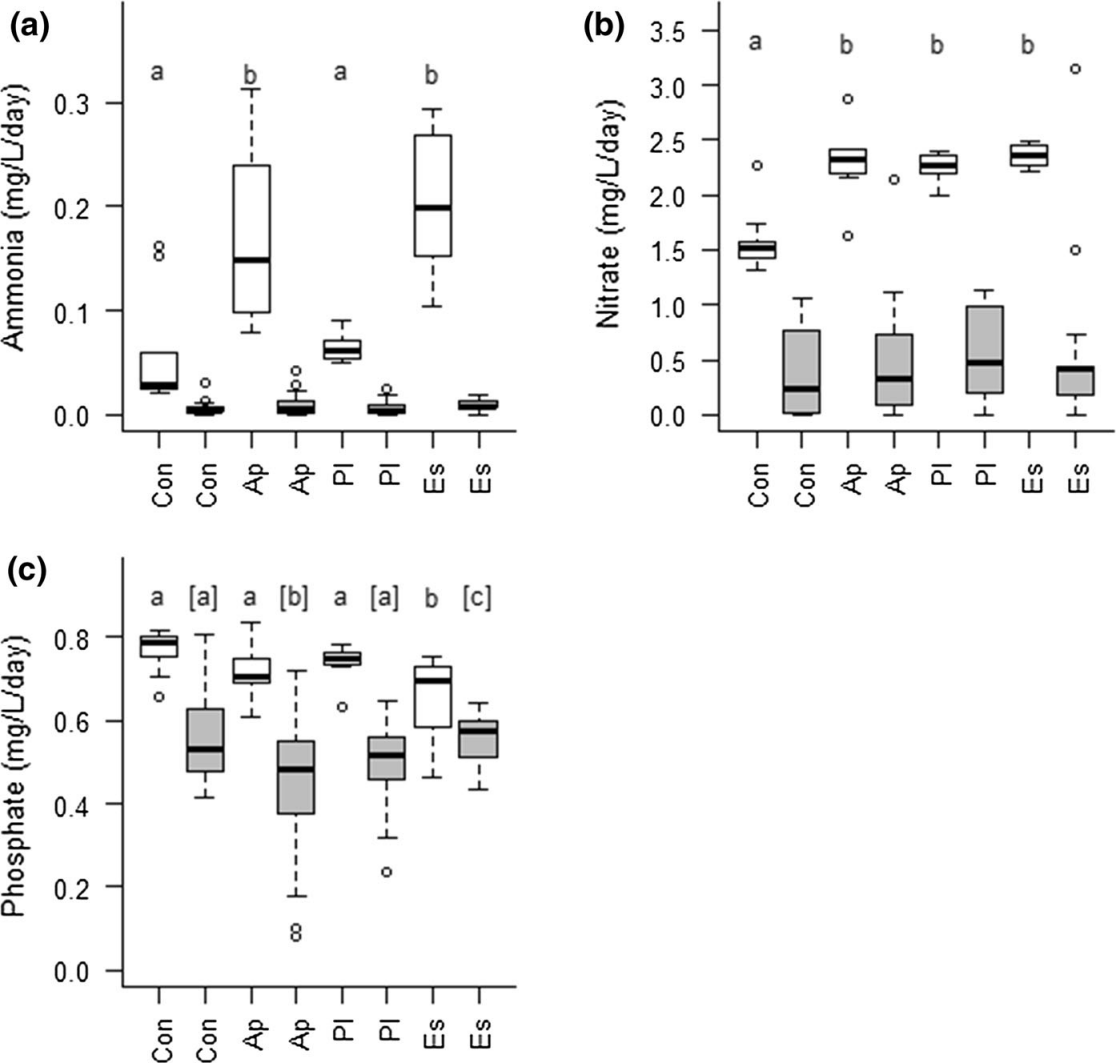
Nutrient production rates for incubations without leaf litter and biofilms (white bars) and for the main experiment in the presence of leaf litter and biofilms (grey bars). Letters above boxplots indicate groupings based on post hoc tests, with those in parentheses distinguishing the detritivory experiment and those without parentheses for the incubation for excretal products. Abbreviations of x-axis and letters above box-plots as per Fig. 1

Dissolved nutrients (ammonia, nitrate and phosphate) did not differ among treatments, though ammonia and phosphate concentrations were correlated with decapod mass (Table 1). These negligible differences in nutrient concentration among treatments following detritivory contrast to the differences in nutrient concentrations among decapod species in the absence of biofilms (Table 2; Fig. 3). Both *A. pallipes* and *E. sinensis* treatments had higher concentrations of ammonia than *P. leniusculus*, which were similar to decapod-free controls (Fig. 3a). Nitrate concentrations were elevated in all decapod species compared to controls (Fig. 3b). Phosphate concentration was significantly lower for *E. sinensis* compared to the other decapod species (Fig. 3c). Species identity considered as the only factor produced the best performing model for ammonia and phosphate, with differences in AICs for competing factors in nitrate negligible (Table 2). Nutrient concentrations were higher in controls without leaf litter and biofilm compared to those with, highlighting that nutrients were consumed by microbial consumers of leaf litter/FPOM as well as by the biofilm.

**Table 2:** Generalised linear models for nutrient excretion for native and invasive alien decapod treatments

Response variable	Effects model	*df*	Residual deviance	Pr(> Chi)	AIC
Ammonia (NH_3_-N)	**Species**	**3**	**1.163**	**>** **0.001**	**−** **85**
	Mass	1	0.044	0.006	− 53
	Species * mass	2	0.031	0.512	− 58
Nitrate (NO_3_-N)	Species	3	3.512	> 0.001	4
	Mass	1	0.127	0.078	4
	Species * mass	2	0.102	0.311	0
Soluble reactive phosphorous (PO_4_-P)	**Species**	**3**	**0.082**	**0.011**	**−** **77**
	Mass	1	0.003	0.464	− 50
	Species * mass	2	0.002	0.801	− 47

Models that show lowest AIC value are highlighted in bold

### Biofilm response

Primary productivity and biomass on both established and accrued biofilms did not differ among treatments (Table 1). All tiles used in the experiment showed significantly increased growth of biofilm biomass (AFDM, GLM *p* < 0.001) but not chlorophyll *a* in relation to the tiles unexposed to conditioned leaf litter processing, regardless of treatment (Suppl. Fig. 2). Biofilm mass and chlorophyll *a* showed weakly positive but significant correlation with decomposition rate, nitrate and phosphate (summarised in Suppl. Table 1).

## Discussion

This study has demonstrated differences in organic matter shredding and nutrient generation by native and invasive decapods. The two invasive alien species showed higher leaf litter decomposition rates in relation to native crayfish and controls, with production of higher levels of secondary products: CPOM, FPOM and DOC. Invasive alien crayfish were also found to generate nutrients at a lower rates compared to the native crayfish, but also to the invasive alien crab, indicating non-redundancy for impacts on nutrient cycling. However, biofilm growth was not affected by these differences in resource availability. *Pacifastacus leniusculus* is extirpating the native *A. pallipes* across Europe. The results of this study indicate that this species replacement may alter nutrient cycling in invaded ecosystems. Furthermore, invasive *P. leniusculus* and *E. sinensis* occur in many water courses where no native crayfish were present (such as in Scotland, Crawford et al. 2006) and our results suggest that invasion by these novel shredders could have strong impacts on nutrient cycling and water quality.

Breakdown rates of leaf litter were higher for the invasive alien decapods *P. leniusculus* and *E. sinensis,* being almost double those for native crayfish. This may reflect the higher metabolism of the invasive alien species driving increased resource use (Rosewarne et al. 2014, 2016). Leaf litter breakdown was greater for the invasive alien species even after factoring in the effect of body mass, suggesting a trait-based cause (such as digestive enzymes, Johnston and Freeman 2005) for enhanced decomposition in addition to body size. These observations are consistent with one laboratory study that compared invasive alien to native decapods in Europe, but did not include *A. pallipes* or *E. sinensis* (Dunoyer et al. 2014). Given that *P. leniusculus* and *E. sinensis* also reach higher mass than the native *A. pallipes*, the difference in detrital processing rates could be even greater in the field. In situ, these decapods are omnivorous, consuming detrital leaves, macrophytes and invertebrates, many of which are also shredders (Bondar et al. 2005; Rudnick and Resh 2005). In addition, sub-adults were used in this study owing to their dominance in the population, but ontogenic effects do occur, with adult *A. pallipes* consuming more detritus (Rosewarne et al. 2013). Thus the outcome for detritivory is likely to be complex, with field manipulation needed to fully examine outcomes in multi-trophic and multifunctioning communities.

Smaller fragments of CPOM (1–10 mm) were produced as a result of shredding, with more CPOM produced in the invasive alien decapod treatments. This organic matter is likely to be consumed by other shredders (such as amphipods, MacNeil et al. 2011; Piscart et al. 2011), whilst these are likely to be consumed also by decapods (Dunoyer et al. 2014). Production of FPOM and DOC was higher in the invasive decapod treatments, likely a result of consumption and processing of leaf material (including nutrients, FPOM, DOC) through the decapod gut, as opposed to mechanical breakdown (Montemarano et al. 2005; Carvalho et al. 2016).

Biofilm biomass and primary productivity were not affected by the presence of decapods, despite consumer-specific differences in production of secondary products. This is in contrast to previous studies which found that biofilms exposed to nitrogen-rich excretal products of crayfish had higher primary productivity (Evans-White and Lamberti 2005). However, it is possible that other trophic groups such as absorbers and collectors would be affected by the increased availability of nutrients in the presence of invasive decapods. When nutrient concentrations were measured in water inhabited by decapods in isolation, ammonia (most likely derived from excretion) differed with the lowest concentrations recorded for *P. leniusculus* indicating this species could act as a nitrogen sink (Vanni 2002). Observed differences between *P. leniusculus* and the native crayfish *Cambaroides japonicus* in Japan (Usio et al. 2006) support our findings of inter-specific decapod effects on water chemistry. *Eriocheir sinensis* showed relatively reduced effects on phosphate concentration in microcosms, suggesting a greater phosphorous–nitrogen ratio in the body tissues. While there was no difference between *E. sinensis* and *A. pallipes*, the crab achieves greater adult body size and densities (Rudnick et al. 2005), and greater resource consumption rate (Rosewarne et al. 2016) indicating it could be a phosphorous sink in newly invaded ecosystems. Further work to determine the likely roles in nutrient cycling by these decapod species and possible consequences of invasion would require measurement of: the elemental composition of these three species; change in excreted products in response to food items (detritus, plants, prey) of different elemental composition; and measurement of the difference in release of nutrients following death among each species.

This experiment has shown that both native and alien decapods increase the breakdown of leaf litter into smaller fragments, including coarse and fine fragments, and dissolved carbon. The potential for nutrient cycling was also found to be different among species. This increase in secondary resources however did not affect the productivity of heterotrophic biofilms growing on tiles. Nevertheless, these results indicate that invasive alien decapods are not ecologically redundant when compared with native crayfish, and have the potential to significantly alter detrital and nutrient processes, thus carbon cycling and food web productivity in freshwater ecosystems.

## Electronic supplementary material

Below is the link to the electronic supplementary material.

Supplementary material 1 (DOCX 78 kb)

## References

[CR1] Abelho M (2001). From litterfall to breakdown in streams: a review. Sci World J.

[CR2] Almeida D, Ellis A, England J, Copp GH (2014). Time-series analysis of native and non-native crayfish dynamics in the Thames River Basin (south-eastern England). Aquat Conserv Mar Freshw Ecosyst.

[CR3] Benfield E (2006) Decomposition of leaf material In: Hauer FR, Lamberti GA (eds) Methods in stream ecology Elsevier Inc. Oxford UK, pp 711–720

[CR4] Blackburn TM, Pyšek P, Bacher S, Carlton JT, Duncan RP, Jarošik V, Wilson JR, Richardson DM (2011). A proposed unified framework for biological invasions. Trends Ecol Evol.

[CR5] Bondar CA, Bottriell K, Zeron K, Richardson JS (2005). Does trophic position of the omnivorous signal crayfish (*Pacifastacus leniusculus*) in a stream food web vary with life history stage or density?. Can J Fish Aquat Sci.

[CR6] Burnham KP, Anderson DR (2004). Multimodel inference: understanding AIC and BIC in model selection. Sociol Methods Res.

[CR7] Capps KA, Flecker AS (2013). Invasive aquarium fish transform ecosystem nutrient dynamics. Proc R Soc Lond B Biol Sci.

[CR8] Carvalho F, Pascoal C, Cássio F, Sousa R (2016). Direct and indirect effects of an invasive omnivore crayfish on leaf litter decomposition. Sci Total Environ.

[CR10] Cohen J (1992). A power primer. Psychol Bull.

[CR11] Crawford L, Yeomans WE, Adams CE (2006). The impact of introduced signal crayfish *Pacifastacus leniusculus* on stream invertebrates. Aquat Conserv Mar Freshw Ecosyst.

[CR12] Cummins KW, Petersen RC, Howard FO, Wuycheck JC, Holt VI (1973). The utilization of leaf litter by stream detritivores. Ecology.

[CR13] Dick JT, Alexander ME, Jeschke JM, Ricciardi A, MacIsaac HJ, Robinson TB, Kumschick S, Weyl OL, Dunn AM, Hatcher MJ, Paterson RA, Farnsworth KD, Richardson DM (2014). Advancing impact prediction and hypothesis testing in invasion ecology using a comparative functional response approach. Biol Invasions.

[CR14] Dunoyer L, Dijoux L, Bollache L, Lagrue C (2014). Effects of crayfish on leaf litter breakdown and shredder prey: are native and introduced species functionally redundant?. Biol Invasions.

[CR15] Dyson KE, Bulling MT, Solan M, Hernandez-Milian G, Raffaelli DG, White PC, Paterson DM (2007). Influence of macrofaunal assemblages and environmental heterogeneity on microphytobenthic production in experimental systems. Proc R Soc B Biol Sci.

[CR16] Ehrenfeld JG (2010). Ecosystem consequences of biological invasions. Annu Rev Ecol Evol Syst.

[CR17] Evans-White MA, Lamberti GA (2005). Grazer species effects on epilithon nutrient composition. Freshw Biol.

[CR18] Gallardo B, Clavero M, Sánchez MI, Vilà M (2015). Global ecological impacts of invasive species in aquatic ecosystems. Glob Change Biol.

[CR20] Gessner MO, Chauvet E, Dobson M (1999). A perspective on leaf litter breakdown in streams. Oikos.

[CR21] Gessner MO, Swan CM, Dang CK, McKie BG, Bardgett RD, Wall DH, Hättenschwiler S (2010). Diversity meets decomposition. Trends Ecol Evol.

[CR23] Johnston D, Freeman J (2005). Dietary preference and digestive enzyme activities as indicators of trophic resource utilization by six species of crab. Biol Bull.

[CR24] Kemp D, Birkinshaw N, Peay S, Hiley PD (2003) Reintroducing the White-clawed Crayfish *Austropotamobius pallipes.* In: Conserving Natura 2000 Rivers Conservation Techniques Series No. 1. English Nature Peterborough

[CR25] Kominoski JS, Rosemond AD, Benstead JP, Gulis V, Maerz JC, Manning DW (2014) Low-to-moderate nitrogen and phosphorus concentrations accelerate microbially driven litter breakdown rates. Ecol Appl 10.1890/14-1113.1 26214929

[CR26] Kristiansen G, Hessen DO (1992). Nitrogen and phosphorus excretion from the noble crayfish, *Astacus astacus* L., in relation to food type and temperature. Aquaculture.

[CR27] MacNeil C, Dick J, Platvoet D, Briffa M (2011). Direct and indirect effects of species displacements; the invading amphipod crustacean *Dikerogammarus villosus* can disrupt aquatic ecosystem energy flow and function. J N Am Benthol Soc.

[CR29] Montemarano J, Kershner M, Leff L (2005). Crayfish effects on fine particulate organic matter quality and quantity. Arch Hydrobiol.

[CR30] Moore JW, Carlson SM, Twardochleb LA, Hwan JL, Fox JM, Hayes SA (2012). Trophic tangles through time? Opposing direct and indirect effects of an invasive omnivore on stream ecosystem processes. PLoS ONE.

[CR31] Moorhouse TP, Macdonald DW (2015). Are invasives worse in freshwater than terrestrial ecosystems?. Wiley Interdiscip Rev Water.

[CR33] Piscart C, Mermillod-Blondin F, Maazouzi C, Merigoux S, Marmonier P (2011). Potential impact of invasive amphipods on leaf litter recycling in aquatic ecosystems. Biol Invasions.

[CR52] R Core Team (2014) R: A language and environment for statistical computing. R Foundation for Statistical Computing, Vienna, Austria. http://www.R-project.org/

[CR34] Ramchunder SJ, Brown LE, Holden J, Langton R (2011). Spatial and seasonal variability of peatland stream ecosystems. Ecohydrology.

[CR35] Rogers D, Watson E (2010) Distribution database for crayfish in England and Wales In: Rees M, Nightingale J, Holdich DM (eds) Species survival: securing White-clawed crayfish in a changing environment. Proceedings of a conference held on 16th and 17th November 2010, Bristol, pp 14–22

[CR36] Rosewarne P, Mortimer R, Dunn A (2013). Size-dependent impacts of the endangered white-clawed crayfish (*Austropotamobius pallipes*)(Lereboullet) on the littoral community. Knowl Manag Aquat Ecosyst.

[CR37] Rosewarne PJ, Svendsen JC, Mortimer RJ, Dunn AM (2014). Muddied waters: suspended sediment impacts on gill structure and aerobic scope in an endangered native and an invasive freshwater crayfish. Hydrobiologia.

[CR38] Rosewarne PJ, Mortimer RJ, Newton RJ, Grocock C, Wing CD, Dunn AM (2016). Feeding behaviour, predatory functional responses and trophic interactions of the invasive Chinese mitten crab (*Eriocheir sinensis*) and signal crayfish (*Pacifastacus leniusculus*). Freshw Biol.

[CR40] Rudnick D, Resh V (2005). Stable isotopes, mesocosms and gut content analysis demonstrate trophic differences in two invasive decapod crustacea. Freshw Biol.

[CR41] Rudnick DA, Chan V, Resh VH (2005). Morphology and impacts of the burrows of the Chinese mitten crab, *Eriocheir sinensis* H. Milne Edwards (Decapoda, Grapsoidea), in south San Fransisco Bay, California, USA. Crustaceana.

[CR42] Schofield KA, Pringle CM, Meyer JL, Sutherland AB (2001). The importance of crayfish in the breakdown of rhododendron leaf litter. Freshw Biol.

[CR43] Schuster GA, Taylor CA, Cordeiro J (2010) *Pacifastacus nigrescens* The IUCN Red List of Threatened Species 2010. eT15867A5247659 10.2305/IUCN.UK.2010-3.RLTS.T15867A5247659.en

[CR44] Steinman AD, Lamberti GA, Leavitt P (1996) Biomass and pigments of benthic algae In: Hauer FR, Lamberti GA (eds) Methods in stream ecology, Elsevier Inc. Oxford UK, pp 357–359

[CR45] Strayer DL (2012). Eight questions about invasions and ecosystem functioning. Ecol Lett.

[CR46] Strayer DL, Dudgeon D (2010). Freshwater biodiversity conservation: recent progress and future challenges. J N Am Benthol Soc.

[CR47] Tran TNQ, Jackson MC, Sheath D, Verreycken H, Britton JR (2015). Patterns of trophic niche divergence between invasive and native fishes in wild communities are predictable from mesocosm studies. J Anim Ecol.

[CR48] Twardochleb LA, Olden JD, Larson ER (2013). A global meta-analysis of the ecological impacts of nonnative crayfish. Freshw Sci.

[CR49] Usio N, Suzuki K, Konishi M, Nakano S (2006). Alien vs. endemic crayfish: roles of species identity in ecosystem functioning. Arch Hydrobiol.

[CR50] Vanni MJ (2002). Nutrient cycling by animals in freshwater ecosystems. Annu Rev Ecol Syst.

[CR51] Vanni MJ, Flecker AS, Hood JM, Headworth JL (2002). Stoichiometry of nutrient recycling by vertebrates in a tropical stream: linking species identity and ecosystem processes. Ecol Lett.

