# Supplementary material for: Transformation of detritus by a European native and two invasive alien freshwater decapods
Source: Biol Invasions. 2018 Jan 31;20(7):1799–808. doi: 10.1007/s10530-018-1661-z (PMC6445490; doi:10.1007/s10530-018-1661-z)

**Supplementary material**

| **Supplemental Table 1. Correlates of biofilms and detrital processing** | | | | | | |
| --- | --- | --- | --- | --- | --- | --- |
| Type of biofilm | Response variable | Explanatory factor | d.f. | Deviance residuals | Pr(>Chi) | R^2^ |
|  |  |  |  |  |  |  |
| Colonised tiles | Total biomass | Decomposition rate | 1 | 0.0002 | **0.04** | 0.03 |
|  |  | FPOM | 1 | 0.0001 | 0.08 | n/a |
|  |  | DOC | 1 | 0.0001 | 0.07 | n/a |
|  |  | NH4-N | 1 | 0.00005 | 0.31 | n/a |
|  |  | NO3-N | 1 | 0.00004 | 0.34 | n/a |
|  |  | PO4-P | 1 | 0.00009 | 0.15 | n/a |
|  |  |  |  |  |  |  |
| New biofilm accrual (blank tiles) | Total biomass | Decomposition rate | 1 | 0.00009 | 0.85 | n/a |
|  |  | FPOM | 1 | 0.00001 | 0.93 | n/a |
|  |  | DOC | 1 | 0.00001 | 0.93 | n/a |
|  |  | NH4-N | 1 | 0.00002 | 0.90 | n/a |
|  |  | NO3-N | 1 | 0.01 | **0.002** | 0.04 |
|  |  | PO4-P | 1 | 0.003 | 0.18 | n/a |
|  |  |  |  |  |  |  |
| Colonised tiles | Primary productivity | Decomposition rate | 1 | 0.09 | 0.68 | n/a |
|  |  | FPOM | 1 | 0.16 | 0.59 | n/a |
|  |  | DOC | 1 | 0.01 | 0.89 | n/a |
|  |  | NH4-N | 1 | 0.09 | 0.68 | n/a |
|  |  | NO3-N | 1 | 0.11 | 0.66 | n/a |
|  |  | PO4-P | 1 | 2.07 | 0.05 | 0.03 |
|  |  |  |  |  |  |  |
| New biofilm accrual (blank tiles) | Primary productivity | Decomposition rate | 1 | 0.10 | 0.40 | n/a |
|  |  | FPOM | 1 | 0.009 | 0.81 | n/a |
|  |  | DOC | 1 | 0.02 | 0.72 | n/a |
|  |  | NH4-N | 1 | 0.20 | 0.25 | n/a |
|  |  | NO3-N | 1 | 0.04 | 0.59 | n/a |
|  |  | PO4-P | 1 | 0.37 | 0.12 | n/a |
|  |  |  |  |  |  |  |
|  |  |  |  |  |  |  |

**Supplementary Figure 1.** Distribution of mass of the different decapod species used in the experiment.


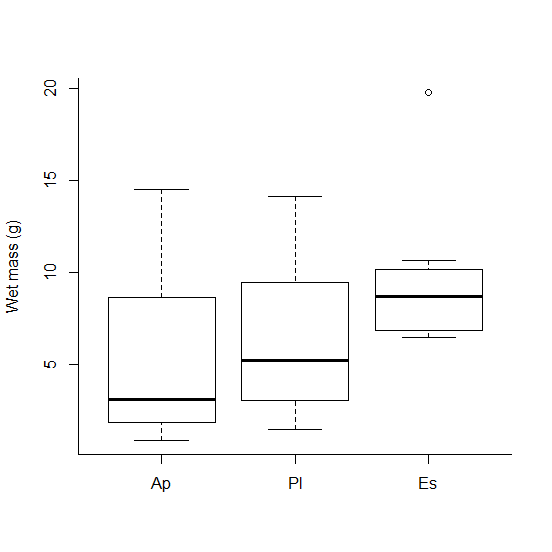


**Supplementary Figure 2.** Biomass accrual (mg ash free dry mass) of established biofilms. Abbreviations as with Fig. 1, with “no treat.” indicating tiles sampled before the experiment to highlight growth.


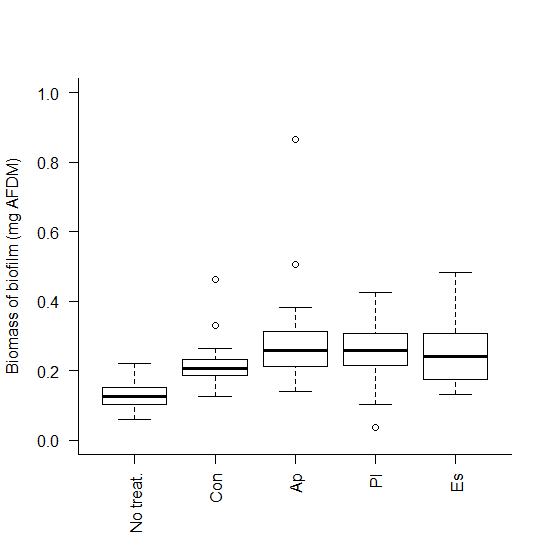


Supplemental Figure 3. Effect sizes (Cohen’s *d*) for variables showing significant relationships with the experimental treatments: a) leaf litter decomposition rate; b) production of CPOM; c) production of FPOM; d) dissolved organic carbon. Abbreviations on the y-axis are the same as those used in Fig.2.


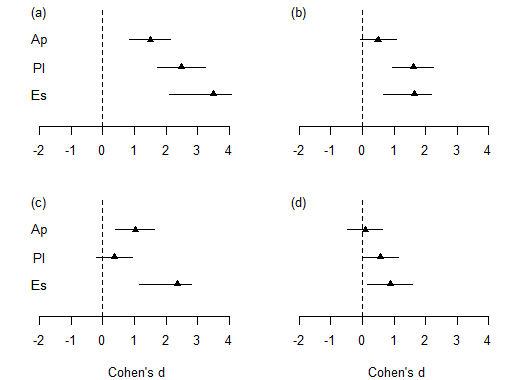


**Supplemental Figure 4. Allometric scaling of leaf litter processing across freshwater decapod species**: Decomposition rates for *Austropotamobius pallipes* (a), *Pacifastacus leniusculus* (b) and *Eriocheir sinensis* (c).


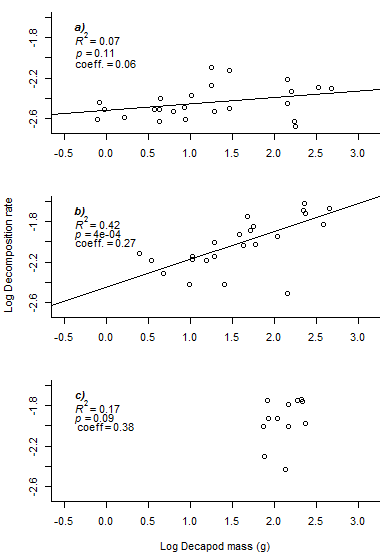


**Supplemental Figure 5. Detritivory performance of native and invasive alien decapod species**. Abbreviations on x-axes: Ap (*A. pallipes*); Pl –invasive alien crayfish (*Pacifastacus leniusculus*); Es –invasive alien crab (*Eriocheir sinensis*).


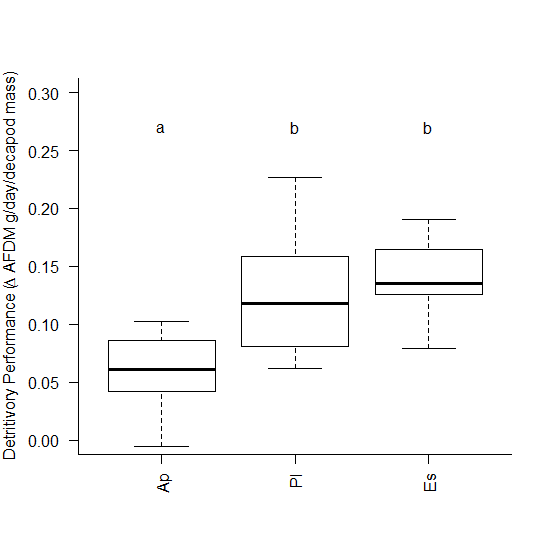

Supplement: Supplementary file 1 — Supplementary material 1 (DOCX 78 kb) [file 10530_2018_1661_MOESM1_ESM.docx]
